# Histomorphological Analysis of Ovarian Neoplasms According to the 2020 WHO Classification of Ovarian Tumors: A Distribution Pattern in a Tertiary Care Center

**DOI:** 10.7759/cureus.38273

**Published:** 2023-04-28

**Authors:** Pallavi Mehra, Sneha Aditi, Krishna M Prasad, Navin k Bariar

**Affiliations:** 1 Pathology and Laboratory Medicine, Patna Medical College, Patna, IND

**Keywords:** 2020 who classification, metastasis, borderline, serous adenocarcinoma, ovarian neoplasm, who 2020

## Abstract

Background: In 2018, ovarian carcinoma ranked as the eighth most common cancer diagnosed and the leading cause of cancer death in women. High-grade serous carcinoma is the most common histological type seen among malignant cases. A diverse group of neoplasms is seen in the ovary with variable clinical, morphological, and histological features, so assessing the nature of ovarian neoplasms further assists in the treatment of the disease.

Aim: This study was conducted to assess the different histopathological variants of ovarian neoplasms according to the latest 2020 World Health Organization (WHO) classification of ovarian tumors. Further analysis of the frequency, age, clinical features in patients, and distribution of various ovarian tumors is assessed.

Materials and methods: A retrospective study was conducted in the Department of Pathology at Patna Medical College and Hospital (PMCH), Patna. The data of the patients from the past three years, from February 2020 to February 2023, were retrieved and assessed. Gross and microscopic findings, including clinical details of patients with ovarian masses, were analyzed from the previous records.

Result: A total of 110 cases of ovarian neoplasms on histopathology were analyzed. The age range was 11-70 years. The types of specimens received were those of total abdominal hysterectomy, salphingoopherectomy, and unilateral or bilateral ovarian cystectomy. The most common presentation was an abdominal mass, followed by pain in the abdomen. The majority of the tumors were benign (69%), malignancy was observed in 24.5% of cases, and borderline tumors were seen in 5.4% of cases. Epithelial tumors were the commonest tumors, accounting for 70%, followed by germ cell tumors (21%). Serous cystadenoma was the commonest benign tumor, followed by mature teratoma and serous cystadenocarcinoma. High-grade serous cystadenocarcinoma was the commonest malignant ovarian tumor (9%), followed by low-grade serous cystadenocarcinoma (4.5%) and metastatic carcinoma of the ovary. Krukenberg tumor was seen in two cases, and a very rare case of sclerosing stromal tumor was seen in one of the cases.

Conclusion: Ovarian neoplasms usually present with a variety of clinicomorphological and histological features. The most common neoplasm observed in the ovary is surface epithelial tumors, which are benign lesions that commonly affect reproductive age groups. Newer advancements like immunohistochemistry (IHC) and genetic studies have made the diagnosis easier and more precise. However, in institutes with limited resources, a histopathological study is still the gold standard in the diagnosis and prognostic evaluation of these tumors.

## Introduction

Two ovaries are situated on either side of the uterus in the pelvis. This is the most common yet complex site for incidents of neoplasm in women [1]. In 2018, ovarian carcinoma ranked as the eighth most common cancer diagnosed and cause of cancer death in women, with an estimated 295,000 cases and 184,000 deaths worldwide. According to the Global Cancer Observatory (GLOBOCAN) 2018, the age-standardized incidence rate for India is 3.8-5.5 cases per 100,000 females per year [2], whereas this was 10.2/100,000 females in India in 2013 [3]. The observed decline was mainly attributed to the use of oral contraceptives, which have long-lasting protective effects against ovarian cancer after several years of use. Other factors, like high parity and breastfeeding, have also been reported as protective factors [1]. The Indian Cancer Registry data project states that the ovary is an important location for carcinoma and comprises up to 8.7% of cancers in various parts of India [4]. However, other factors like obesity, nulliparity, cigarette smoking, and genetic changes pose risk factors for the development of ovarian carcinoma.

About 80% of ovarian tumors are benign and occur in young women between the ages of 20 and 45, whereas 20% are malignant tumors common in older women between the ages of 40 and 65 with poor prognosis [2]. Ovarian cancer has the worst prognosis among gynecological malignancies due to a lack of proper signs, symptoms, and presentation and is usually detected in the later stages of the disease [5]. The precise diagnosis of ovarian carcinoma depends on histopathology before starting definitive treatment.

This study was performed to identify the various histopathological variations in ovarian neoplasm (ON) and assess them according to the World Health Organization's (WHO) 2020 classification. Further analysis of the prevalence, age, clinical features in patients, and distribution of various ovarian tumors is assessed.

## Materials and methods

An observational (retrospective) study was conducted in the Department of Pathology at Patna Medical College and Hospital (PMCH), Patna, India. The data of the patients from the past three years, from February 2020 to February 2023, were retrieved and assessed. A total of 110 cases were analyzed after taking informed consent telephonically from the patient, wherever available. The ethical clearance was granted by Aryabhatta Knowledge University, Patna, India (approval number: 280/22). Inclusion criteria included all the ovarian biopsies and ovarian lesions that were radiologically assayed as either neoplastic or non-neoplastic and received either as a single lesion or as a total hysterectomy. Normal ovaries were excluded from the study.

Gross and microscopic findings, including clinical details of patients with ovarian masses, were analyzed. The ovarian specimens were fixed in 10% neutral buffered formalin. The weight of the tumor was measured. A gross examination was done, visualizing the outer surface and on-cut surface diligently looking for a cyst, its locularity, and type of cystic fluid, further looking for solid areas, papillary projections, hemorrhage, and necrosis. Standard procedures were followed during tissue processing, and paraffin-embedded blocks were made. Tissue sections of 5 μ thickness were cut using a rotary microtome and stained with hematoxylin and eosin, followed by microscopic examination.

The data obtained after microscopy included information about the type of tumor and other relevant details. The data were entered into an Excel sheet, and further analysis was done using IBM Statistical Package for Social Sciences (SPSS) for Windows, Version 25.0, Armonk, NY, to assay the frequencies and percentages of all variables that were documented. The data thus analyzed were correlated for relevant findings and classified according to the 2020 WHO classification of ovarian tumors.

## Results

**Figure 1:**
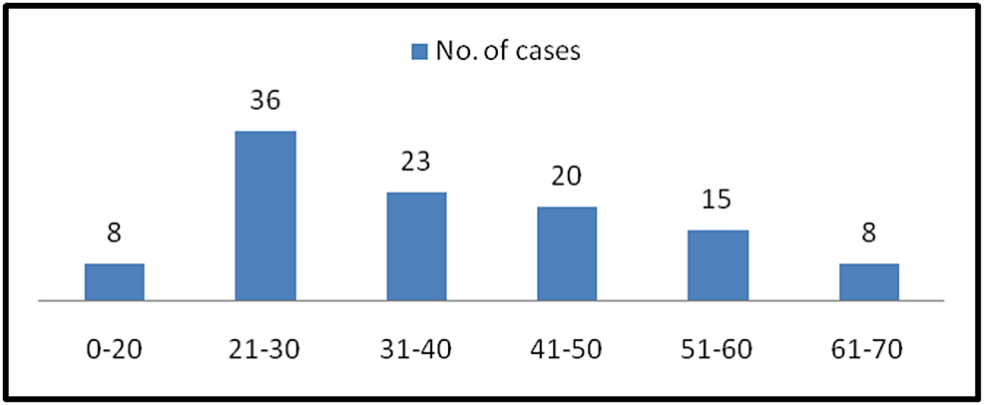
Distribution of age and number of cases of ovarian neoplasms

An observational study was conducted, and a total of 110 cases of ovarian neoplasms were assessed for the patients who underwent ovarian biopsy or hysterectomy. The types of specimens received were those of total abdominal hysterectomy, salphingoopherectomy, and unilateral or bilateral ovarian cystectomy. The maximum number of patients were in their third decade of life, and the distribution of the cases was within the age range of 11-70 years, as shown in Figure 1. The majority of cases of benign tumors were mostly seen in 21-30-year-olds, borderline tumors in 61-70-year-olds, and malignant tumors in the 41-50-year-old age group.

**Figure 2:**
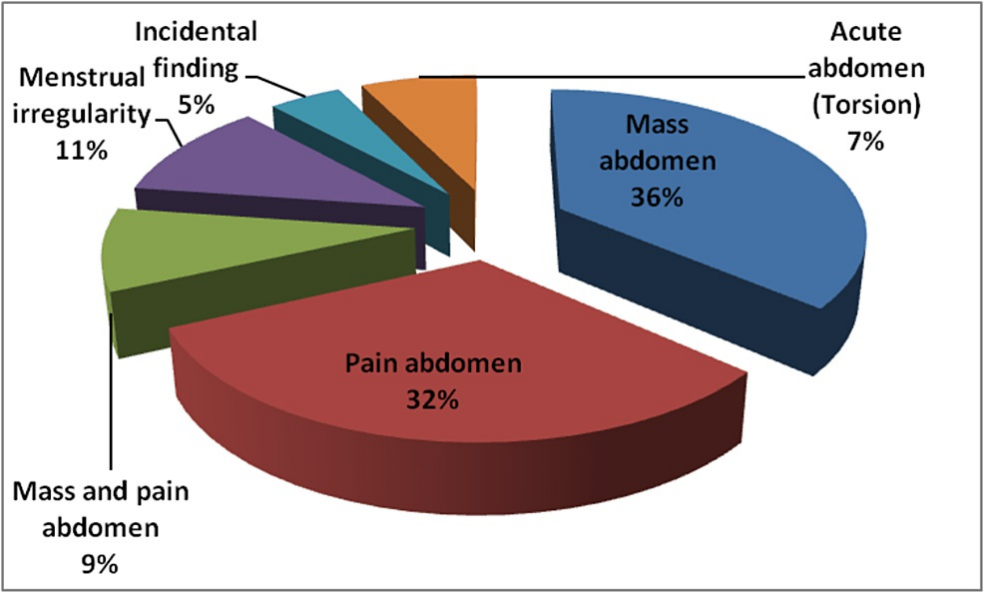
Clinical manifestations of patients with ovarian neoplasms

The most common presentation of ovarian neoplasms was mass and heaviness in the abdomen, seen in 36% of patients, followed by abdominal pain (32%). Few tumors presented with torsion and came with acute findings of distress and pain; this was seen in 7% of cases, as shown in Figure 2. An emergency laparotomy was done in such patients, and a sample was sent for histopathological examination.

Out of 110 cases of ovarian neoplasms, 69% were benign; malignancy was seen in 24.5% of cases, while borderline tumors were seen in 5.4% of cases. These tumors were classified according to WHO classification 2020 and categorized as shown in Table 1. Surface epithelial tumors constituted the majority of the ovarian neoplasm with 77 cases, followed by germ cell tumors, which constituted 23 cases; sex cord stromal tumors were seen in three cases; and metastatic tumors were found in seven cases.

Among benign tumors, serous cystadenomas were seen in the maximum number of cases, constituting 38% of the total cases, which were surface epithelial tumors. These were followed by benign teratomas, constituting 16.3% of the total cases; germ cell tumors; and mucinous cystadenomas, accounting for 7.2% of total ovarian neoplasms. Among malignant lesions, high-grade serous cyst adenocarcinomas were seen in 9% of cases, followed by low-grade serous cystadenocarcinomas (4.5%).

Seven occurrences of metastatic carcinoma were seen, including two cases of signet ring cell adenocarcinoma (Krukenberg tumor), two cases of undifferentiated adenocarcinoma, and two cases of squamous cell carcinoma (SCC) in individuals who had previously experienced cervical SCC.

A sclerosing stromal tumor, one of the rare tumors of the ovary, was also found in a 24-year-old patient, where the tumor weighed around 2kg and was 20cm in length.

**Table 1:** Ovarian neoplasm distribution according to WHO Classification (2020) NOS: not otherwise specified; SCC: squamous cell carcinoma

Histopathological diagnosis	Nature of tumor	Types	Number of cases (N=110)	Percentage (100%)
Surface epithelial neoplasm (n=77)	Serous	Serous cystadenoma NOS	42	38%
		Serous borderline tumor NOS	4	3.6%
		Low-grade serous carcinoma	5	4.5%
		High-grade serous carcinoma	10	9%
	Mucinous	Mucinous cystadenoma	8	7.2%
		Mucinous borderline tumor	2	1.8%
		Mucinous adenocarcinoma	3	2.7%
	Endometroid	Endometroid cystadenoma NOS	0	0
		Endometroid tumor, borderline	0	0
		Endomtroid adenocarcinoma NOS	0	0
		Seromucinous carcinoma	0	0
	Clear cell	Clear cell cystadenoma	0	0
		Clear cell borderline tumor	0	0
		Clear cell adenocarcinoma NOS	0	0
	Seromucinous	Seromucinous cystadenoma	2	1.8%
		Seromucinous adenofibroma	0	0
		Seromucinous borderline	0	0
	Brenner	Brenner tumor NOS	1	1%
		Brenner tumor, borderline malignancy	0	0
		Brenner tumor, malignant	0	0
Sex cord stromal tumors (n=3)	Pure stromal tumors	Fibroma NOS	1	1%
		Thecoma NOS	1	1%
		Sclerosing stromal tumor	1	1%
		Fibrosarcoma	0	0
	Pure sex cord tumors	Adult granulosa cell tumor	0	0
		Granulose cell tumor, juvenile	0	0
		Sertoli cell tumor NOS	0	0
	Mixed-sex cord stromal tumors	Sertoli leydig cell tumor NOS	0	0
Germ cell tumors (n=23)		Teratoma benign	18	16.3%
		Immature teratoma	0	0
		Dysgerminoma	3	2.7%
		Yolk sac tumor NOS	1	1%
		Embryonal carcinoma NOS	0	0
		Mixed germ cell tumor	0	0
		Struma ovarii NOS	1	1%
Other carcinomas (n=7)		Signet ring cell adenocarcinoma	2	1.8%
		Metastatic carcinoma-SCC	2	1.8%
		Metastatic adenocarcinoma	2	1.8%
		Carcinoma undifferentiated	1	1%

## Discussion

Ovarian neoplasms are one of the most notorious tumors in the female body. Because of their anatomical position, they remain undiagnosed for an extensive period of time. An ovarian neoplasm causes mass in the abdomen, pain, and abdominal distension in many cases and hence presents as a silent killer. Due to monthly endocrine and traumatic insults during ovulatory cycles, the ovaries have become susceptible to tumor genesis [3].

**Figure 3:**
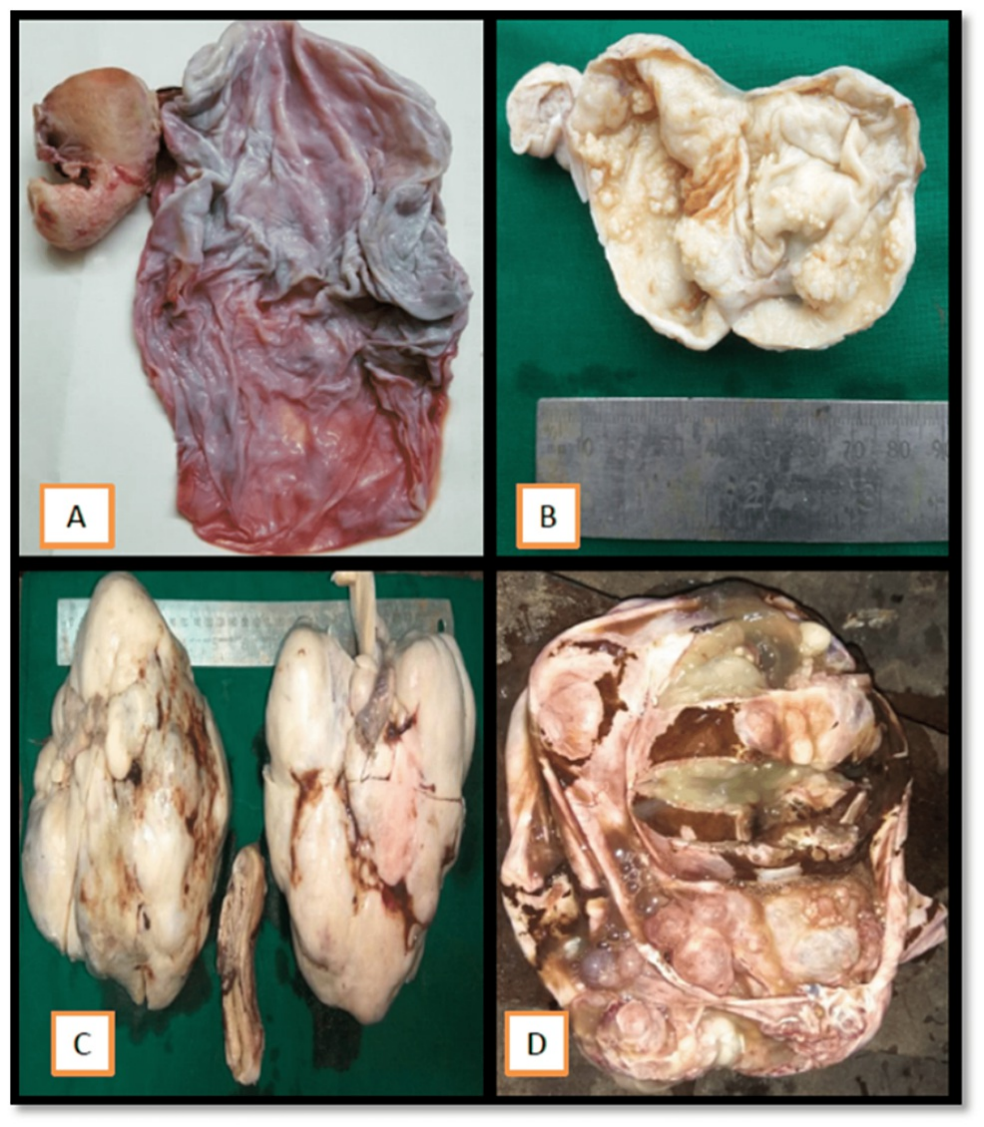
Gross picture of surface epithelial tumors: A) Mucinous cystadenoma; B) Borderline serous cystadenoma; C) Serous cystadenocarcinoma; D) Mucionus cystadenocarcinoma

**Figure 4:**
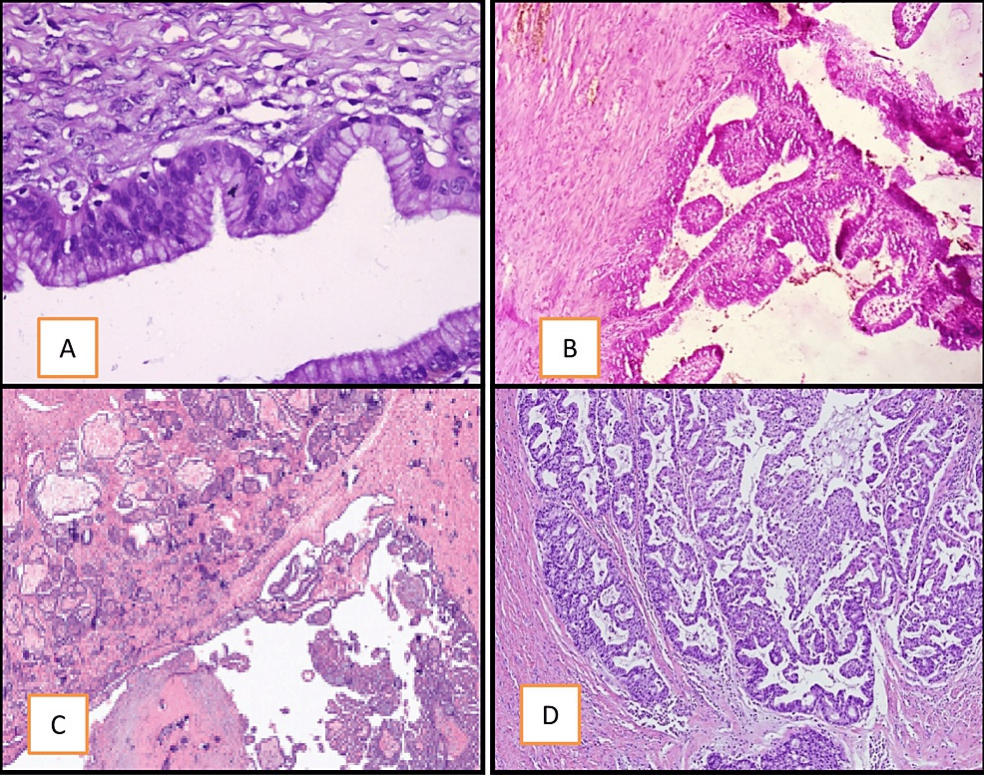
Microscopy of a surface epithelial tumor: A) Mucinous Cystadenoma; B) Borderline Serous Cystadenoma; C) Mucinous Cystadenocarcinoma; D) Serous Cystadenocarcinoma

Ovarian neoplasms were categorized according to the 2020 WHO classification of ovarian tumors into serous, mucinous, endometroid, clear cell, seromucinous, and Brenners; these are surface epithelial tumors (SET) categorized into benign, borderline, and malignant categories [2]. A few of the SETs gross pictures and photomicroscopy are shown in Figure 3 and Figure 4. In our study, surface epithelial tumors were seen in the majority of the cases, accounting for 69%, whereas serous cystadenoma was seen maximally in 38% of the cases.

**Figure 5:**
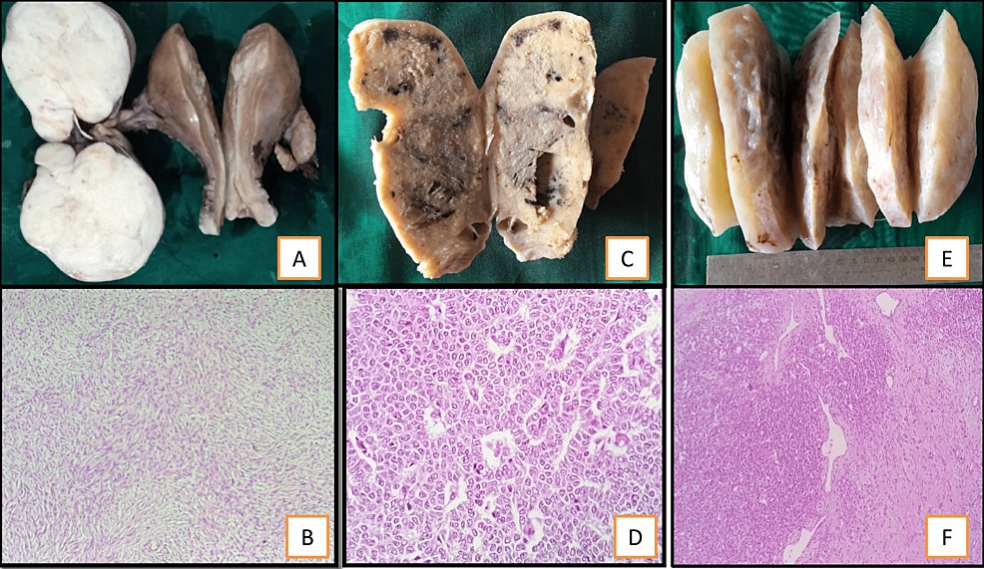
Gross and microscopic exams of SSTs: A and B) Fibroma; C and D) Granulosa cell tumor; E and F) Sclerosing stromal tumor SSTs- Sex cord stromal tumors

Sex cord stromal tumors (SSTs) are categorized into pure stromal tumors, pure sex cord tumors, and mixed sex cord stromal tumors. SSTs include fibromas, thecomas, sclerosing stromal tumors, juvenile granulosa cell tumors, adult granulosa cell tumors, and Sertoli-Leydig cell tumors, which are commonly found. SSTs are gross, and their microscopy is described in Figure 5. In our study, SSTs were seen in four cases. A sclerosing stromal tumor was seen in one of our cases, which is a rare tumor seen in only 2%-5% of cases.

**Figure 6:**
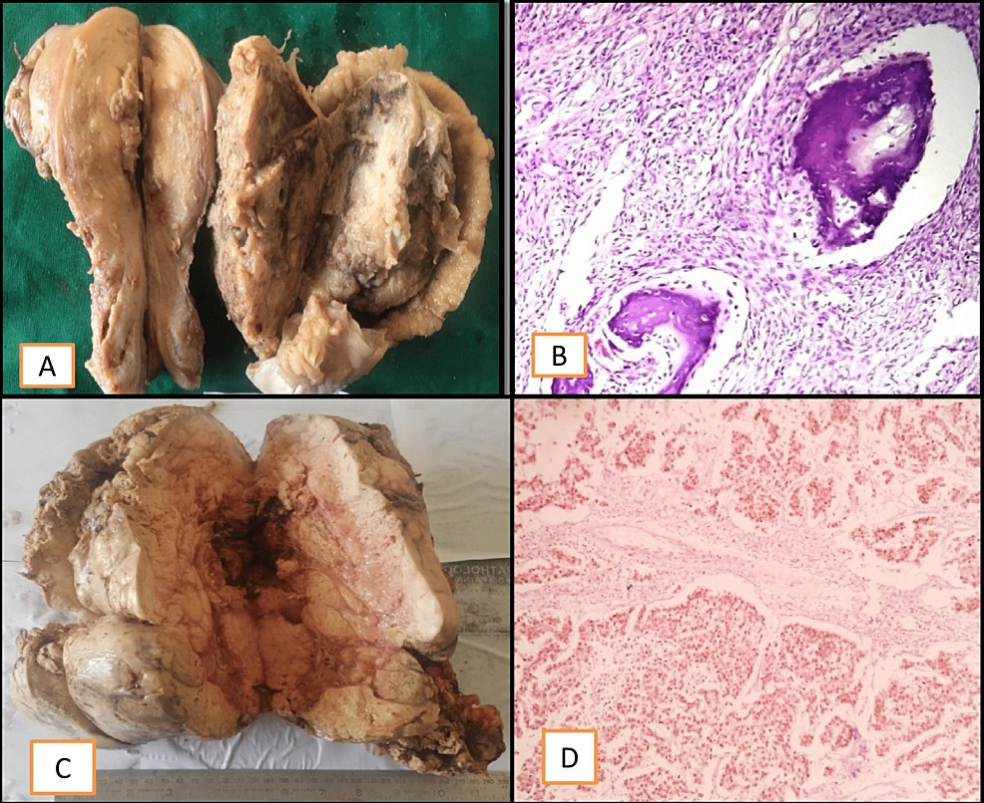
Germ cell tumor: A and B) Gross and microscopy of a mature cystic teratoma; C) Gross picture of Dysgerminoma; D) IHC photomicrography of dysgerminoma showing Oct3/4 positivity

Germ cell tumors (GCT) comprise mature cystic teratomas, immature teratomas, dysgerminomas, yolk sac tumors, embryonal carcinomas, choriocarcinomas, mixed germ cell tumors, monodermal teratomas (struma ovarii and strumal carcinoids), and gonadoblastomas. A few GCTs are described in Figure 6. In our study, germ cell tumors were seen in 23 cases; teratoma was the most common germ cell tumor, accounting for 16% of the cases. Three cases of dysgerminoma were seen in our study, which were proven by IHC, where octamer-binding transcription factor (OCT) 3/4 positivity was seen, as shown in Figure 6D.

**Figure 7:**
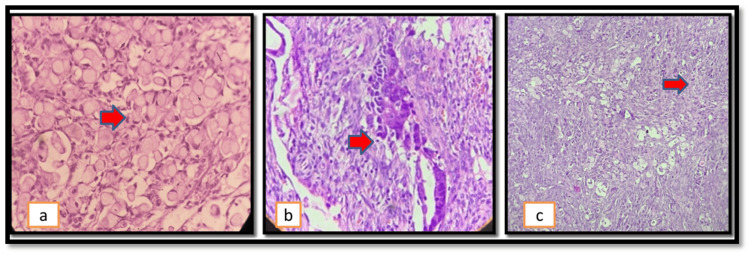
Metastatic carcinoma: a) signet ring cell adenocarcinoma; the red arrow shows signet rings; b and c) metastatic adenocarcinoma; the red arrow shows the deposits

Other tumor types are mesenchymal tumors, other carcinomas, and metastases. Seven cases of metastasis were seen in our study. The most common metastasis seen was adenocarcinoma, as shown in Figure 7. Mesonephric cancers like adenocarcinomas are a newer addition to the 2020 WHO classification, along with the category of mixed carcinomas [2].

We included 110 cases of ovarian tumors in our study. The majority of cases were seen in the third decade of life, with an age range of 11-70 years. This was in contrast with the studies by Poonam et al. [6], Sampurna et al. [7], and Thakkar N et al. [8], where the majority of the cases were in the fourth to fifth decade of life, but our study was in accordance with the study by Batool et al. [5], where most of the women affected were in the age range of 21-30. Pain in the abdomen and mass per abdomen are the most common clinical presentations for ovarian neoplasms. This was in accordance with Kaur A et al.'s [4] findings; Amita S Patel et al. [1] stated in their study that the majority of the patients presented with vague abdominal pain.

When the comparison was made among different types of percentage incidence of ovarian neoplasm, it was found that our study showed the maximum number of benign cases followed by malignant and borderline tumors, which was in concordance with the studies by Poonam et al. [6], Singh et al., and many others as described in Table 2, but was not correlating with Gupta N et al. as they showed the predominance of borderline tumors compared with malignant ones.

**Table 2:** Comparison of the percentage incidence of ovarian tumors in various studies

Authors		Benign tumors	Borderline tumors		Malignant tumors
Amita S Patel et al. [1]		93.2%	0.6%		6.2%
Asmeet Kaur et al. [4]		73.9%	3.6%		22.4%
Batool et al. [5]		80.2%	2.82%		14.61%
Poonam Sharma et al. [6]		89.6%	3.6%		9.8%
Sampurna et al. [7]		66%	3.5%		30.5%
Thakkar N et al. [8]		84.5%	2.3%		13.2%
Sawant A et al. [9]		75.7%	6.6%		18.2%
Singh S et al. [10]		80.8%	1.6%		20%
Hathila et al. [11]		62.3%	4.4%		33.3%
Maheshwari V et al. [12]	71.7%		4.4%	23.7%	
Gupta N et al. [13]	72.9%		22.9	4.2%	
Pilli GS et al. [14]	76%		2.8%	21%	
Badge A et al. [15]	74%		5%	21%	
Present study	69%		5.4%	24.5%	

When a comparison was made between different histopathological types, it was found that the most common neoplasm was a surface epithelial tumor, followed by a germ cell tumor, which was maximally correlating with other studies as shown in Table 3. However, in our study, metastasis was seen in a greater number of cases compared to sex cord stromal tumors.

**Table 3:** Comparison of different histopathological types of ovarian tumors

Authors	Surface epithelial tumors	Germ cell tumor	Sex cord stromal tumors	Others (metastasis)
Poonam Sharma et al. [6]	69.6%	25.8%	4.1%	0
Singh S et al. [10]	69.1%	25.8%	4.1%	0
Sampurna et al. [7]	66%	3.5%	30.5%	2%
Batool et al. [5]	63%	29%	6%	0.5%
Hathila et al. [8]	76.7%	13.03%	10%	0
Sawant A et al. [9]	84.8%	9.1%	6.1%	0.0
Amita S Patel et al. [2]	77.7%	18.5%	3.8%	0
Gupta N et al. [14]	65.6%	23.9%	8.3%	0
Pilli GS et al. [15]	71%	21%	7%	0.7%
Present study	70%	20.9%	2.7%	6.4%

The limitation in this study was the sample size, as a larger sample size would have helped in categorizing a large number of cases. This was just the study of a single institute; hence, it does not consider the frequency of ovarian neoplasms in a whole country or state.

## Conclusions

The overall diagnosis of ovarian neoplasms primarily depends on the histomorphological examination, which is still the gold standard method of evaluation of the tumor; however, the support of ancillary studies such as special stains and immunohistochemistry is always helpful in the exact categorization of the neoplasm. Clinical parameters such as age, size, site, and stage are additional factors that guide the overall management and prognosis of this neoplasm. It is concluded that benign ovarian neoplasms are found more frequently, followed by malignant ones. Surface epithelial tumors are the most common histological subtype of ovarian tumors. Thus, categorizing ovarian tumors according to histopathological features as described in the 2020 WHO classification of ovarian tumors into various categories helps to know the clinical presentation, treatment, clinical outcome, and prognosis of the tumors.
